# Cross−national comparison of major depressive disorder burden in China, India, and the United States of America: an age−period−cohort analysis of GBD 2021

**DOI:** 10.3389/fpsyt.2025.1686919

**Published:** 2026-01-20

**Authors:** Chengcheng Zhang, Chun Zhang, Xiaotong Hong, Yifeng Xiao, Wenjuan Ying, Wei Ma

**Affiliations:** 1 Institute of Nursing Research, The First Affiliated Hospital of Shantou University Medical College, Shantou, Guangdong, China; 2 Department of Nursing, Shantou University Medical College, Shantou, Guangdong, China; 3 Preventive Medicine Center, Yulin Hospital of Traditional Chinese Medicine, Yulin, Shanxi, China

**Keywords:** cross-national comparison, disease burden, GBD, major depressive disorder, risk factors, trends

## Abstract

**Background:**

Major depressive disorder (MDD) poses a significant global public health challenge, contributing substantially to disability and mortality. Despite its widespread impact, the burden of MDD varies considerably across different countries due to distinct sociocultural, economic, and healthcare contexts. This study compares the burden of MDD in China, India, and the United States of America from 1990 to 2021, employing a cross-national age-period-cohort analysis using data from the Global Burden of Disease (GBD) 2021 study. We also project future trends in MDD burden up to 2035.

**Methods:**

Data on MDD prevalence, years lived with disability (YLDs), and age-standardized prevalence rates (ASPR) and age-standardized YLD rates (ASYR) were obtained from the GBD 2021 database. We employed statistical methods including Joinpoint regression, Bayesian age-period-cohort (BAPC) modeling, and the calculation of the average annual percentage change (AAPC) to analyze trends and forecast future MDD burden across the three countries.

**Results:**

Between 1990 and 2021, the number of MDD cases increased in all three countries, with marked variations in age-standardized rates. In China, ASPR and ASYR showed modest declines, with a notable decrease in the younger age groups, while older adults, particularly those aged 70-74, experienced rising rates. India demonstrated more stability in age-standardized rates, while the United States of America saw a pronounced increase in both prevalence and YLDs. Gender disparities were evident in all countries, with females consistently bearing a higher burden than men. Projections for 2035 suggest that the MDD burden will continue to rise in the United States, while trends in China are expected to slightly decrease and remain relatively stable in India.

**Conclusions:**

The escalating burden of MDD, particularly in the United States of America, highlights the urgent need for tailored interventions that address the specific demographic and sociocultural factors influencing mental health in each country. In China and India, the growing absolute number of MDD cases calls for strengthened mental health services, especially for older adults and women, while in the United States of America, tackling rising prevalence among younger populations and addressing underlying socioeconomic inequalities will be crucial. Implementing comprehensive mental health strategies, including improved access to care and targeted preventive interventions, are essential to alleviate the future impact of MDD in these countries.

## Introduction

Major depressive disorder (MDD) is a leading cause of disability worldwide, associated with substantial morbidity, mortality, and societal costs, and affects up to 20% of the population (1). Symptoms of MDD include depressed mood, anhedonia (loss of interest or pleasure), changes in appetite, fatigue, and impaired concentration (2). These symptoms significantly impair daily functioning and are associated with an increased risk of comorbid conditions, such as anxiety disorders and cardiovascular diseases (3). Furthermore, individuals with MDD often experience physical comorbidities, including obesity and metabolic disorders, which exacerbate the overall disease burden (4). Severe MDD is also linked to suicidal ideation or behavior, placing a substantial public health and economic burden on society and patients’ families (5). However, a significant proportion of individuals with MDD do not achieve full symptom relief with first-line treatments, leading to prolonged disability and a greater burden on healthcare systems (6). Amid the post-COVID-19 global recession, increasing pressures related to health, livelihood, and education further highlight the need for urgent public health interventions for the prevention and control of MDD. Epidemiological research plays a crucial role in elucidating the burden of MDD, providing essential insights into its determinants and guiding the development of preventive and therapeutic strategies.

The Global Burden of Disease Study (GBD), coordinated by the Institute for Health Metrics and Evaluation (IHME), systematically collects and analyzes data on the incidence, prevalence, and burden of diseases, including mental health disorders. The 2021 GBD study ranked depressive disorders, including MDD, among the most burdensome non-fatal conditions worldwide, with 56.33 million DALYs and over 332 million people affected (7). Specifically, MDD has become a growing public health issue in countries with rapid socio-economic development, such as China and India, as well as in developed nations like the United States, each facing unique challenges in addressing the disorder.

This study uses the GBD 2021 database to evaluate the epidemiological characteristics of MDD in China, India, and the United States, selected for their distinct differences in population size, economic development, healthcare systems, and cultural contexts. In China, MDD is influenced by cultural stigma and traditional values that emphasize emotional restraint, while in India, perceptions of mental illness are shaped by religious beliefs and family norms, often viewed through a moral or personal lens (8, 9). In the United States, MDD is linked to individualistic social structures, higher social isolation, and economic inequality (10). The comparison of MDD burden across these three countries reveals key epidemiological patterns shaped by distinct socioeconomic contexts, providing valuable insights for public health policies, medical research, and global mental health strategies. Building on these insights, we also project future trends in MDD burden. These projections are essential for health-policy planning, as they help anticipate future service needs, guide resource allocation, and inform the development of targeted prevention and intervention strategies for at-risk populations.

## Methods

### Overview

This analysis utilizes data from the GBD 2021 study, which includes 371 diseases, injuries, and 88 risk factors across 204 countries and territories (11). All epidemiological estimates were extracted using the interactive GBD Results Tool. As this study exclusively employs publicly available, aggregated population-level data, it did not require ethics committee approval.

### Definition of MDD

MDD was defined according to International Classification of Diseases Tenth Edition (ICD-10) and the Diagnostic and Statistical Manual of Mental Disorders Fifth Edition (DSM-V), characterized by at least one major depressive episode lasting at least two weeks with symptoms of depressed mood or loss of interest (12).

### Data source

Data for this study were extracted from the GBD Results Tool, accessible via the GBD database website (https://ghdx.healthdata.org/gbd-2021). The estimates were retrieved stratified by sex, age, and location. Prevalence data from 1990 to 2021 were extracted using the GBD Results Tool, setting parameters for ‘Major depressive disorder’, ‘Prevalence’, and ‘Number’ and ‘Rate’. YLDs, quantifying non-fatal health loss, were similarly obtained using the same tool by setting ‘Measure’ to ‘YLDs’. Given the potential impact of global events, such as the COVID-19 pandemic, on mental health, certain years may represent outliers due to pandemic-related factors. These years were not excluded from the analysis, and any significant deviations from expected trends will be addressed in the results section.

### Data stratification

The analysis focused on three countries: China, India, and the United States of America. These countries were selected for their differences in cultural contexts, economic development, healthcare systems, and demographic profiles, which allow for a comprehensive understanding of MDD burden in diverse socioeconomic settings. The population was stratified by age and sex. Age was categorized in 5-year intervals from 0 to 95 years (<5, 5–9, 10–14, …, 90–94, ≥95 years). Sex was classified as female and male.

### Burden quantification indicators

To quantify trends in the prevalence and YLDs of MDD, we used age-standardized prevalence rates (ASPR) and age-standardized YLD rates (ASYR), calculated per 100,000 individuals, and estimated annual percentage change (EAPC). Prevalence was defined as the total number of individuals living with MDD a given period, including both new and pre-existing cases. Using GBD 2021 data, we calculated prevalence as the number of affected individuals per 100,000 population. YLDs quantify the burden of living with a nonfatal condition, adjusted for disease severity. It is calculated using the following formula: YLDs = Number of Cases × Disability Weight (DW) × Duration of the Condition. Here, the disability weight (DW) reflects the impact of the condition on an individual’s quality of life, ranging from 0 (perfect health) to 1 (death). We analyzed temporal trends using a linear regression model with the natural logarithm of the age-standardized rate (ASR, representing either ASPR or ASYR) as the dependent variable and the calendar year as the independent variable: y=α+βx+ϵ, where y=In (ASR) and x represents the calendar year. The EAPC was calculated from the β coefficient using the formula: EAPC = 100 × (exp(β) − 1) (13). An EAPC greater than zero indicates an increasing trend in age-standardized indicators, while an EAPC less than zero indicates a decreasing trend. A constant trend is indicated when the 95% confidence intervals (CIs) of the EAPC include 0.

### Statistical analysis

#### Disease burden estimates

Disease burden estimates, including prevalence, YLDs, ASPR, and ASYR, are reported with their corresponding 95% uncertainty intervals (UIs), reflecting the variability and inherent uncertainty in the epidemiological estimates. Trend estimates (EAPC) are presented with 95% confidence intervals (CIs), reflecting the variability in trend estimates. These indicators were visualized using bar charts, stratified by age, sex, and location. Joinpoint regression identified significant inflection points in trends over time, calculating the annual percentage change (APC) for each linear segment and the average annual percentage change (AAPC) over the entire period. An increasing trend was concluded if the lower bound of the AAPC’ s 95% CI was greater than zero; a decreasing trend was concluded if the upper bound was less than zero. If neither condition was met, the ASR was considered stable.

#### Bayesian age-period-cohort forecasting model

For forecasting, we employed the Bayesian age-period-cohort (BAPC) model (14) using integrated nested Laplace approximations (INLA) for Bayesian inference. The model assumed that age, period, and cohort effects were similarly influential. Priors were smoothed using a second-order random walk, with normal distributions parameterized by the 95% uncertainty intervals (UIs) from GBD 2021 data. This approach accounted for data variability in the projections. To propagate uncertainty, these normal distributions, parameterized by the UIs’ standard deviations, ensured that the projections reflected variability in the data rather than relying on point estimates (15). Model validity was assessed through posterior predictive checks by comparing forecasted and observed values. Sensitivity analyses were performed by varying prior assumptions (e.g., standard deviations) to evaluate the stability of the forecasts.

### Data analysis and statistical approach

All analyses and visualizations were performed using R software (version 4.3.1). The ‘JD_GBDR’ package (version 2.3.3; Jingding Medical Technology Co., Ltd., Beijing, China) was used for specific burden of disease calculations. Statistical significance was defined as a two-sided p value <0.05.

## Results

### National level prevalence and YLDs of MDD

The prevalence of MDD increased across the three countries, but the ASPR showed divergent trends. In China, the number of cases rose, but the ASPR decreased by –12.06% (EAPC = –0.62, 95% CI: –0.80 to –0.44). In India, the number of cases increased with a slight rise in the ASPR (4.39%, EAPC = –0.88, 95% CI: –1.22 to –0.54). The United States of America exhibited the most pronounced increase, with a 75.11% rise in ASPR (EAPC = 0.88, 95% CI: 0.53 to 1.24) (**Table 1**).

**Table 1:** Prevalence and age-standardized rates (ASR) of major depressive disorder (MDD) in 1990 and 2021, and Trends from 1990 to 2021.

Location	1990 (95%UI)	2021 (95%UI)	1990 ASR per 100000 No.(95%UI)	2021ASR per 100000 No.(95%UI)	Percentage change (100%)	1990–2021 EAPC (95% UI)
China	18,795,495.53 (16,357,951.13, 22,103,729.49)	25,999,756.81 (22,535,010.18, 30,187,158.57)	1,622.13 (1,427.65, 1,880.26)	1,426.49 (1,241.64, 1,653.11)	-12.06 (-17.39, -5.71)	-0.62 (-0.80, -0.44)
India	20,916,364.99 (18,055,537.89, 24917393.26)	45,409,102.29 (39,275,219.46, 53,483,160.87)	3,048.31 (2,664.72, 3,579.87)	3,182.03 (2,765.60, 3,725.98)	4.39 (-0.55, 9.85)	-0.88 (-1.22, -0.54)
United States of America	6,875,709.34 (6,091,175.89, 7,959,034.73)	15,061,213.75 (13,368,893.14, 17,190,652.21)	2,541.98 (2,250.24, 2,938.99)	4,451.34 (3,918.34, 5,099.46)	75.11 (66.38, 84.46)	0.88 (0.53, 1.24)

ASR, Age-Standardized Rate; EAPC, Estimated Annual Percentage Change; UI, Uncertainty Intervals. The values represent the number of cases of Major Depressive Disorder (MDD) in 1990 and 2021, along with the corresponding ASR per 100,000 and the percentage change from 1990 to 2021, including the EAPC with 95% uncertainty intervals.

The trends in ASYR varied across the three countries. In China, the ASYR decreased by –12.95% (EAPC = –0.65, 95% CI: –0.83 to –0.48), while in India, it increased by 5.01% (EAPC = –0.86, 95% CI: –1.20 to –0.52). The United States of America experienced the largest increase, with the ASYR rising by 74.26% (EAPC = 0.87, 95% CI: 0.52 to 1.23) (**Table 2**).

**Table 2:** YLDs and age-standardized rates (ASR) of major depressive disorder (MDD) in 1990 and 2021, and trends from 1990 to 2021.

Location	1990 (95%UI)	2021 (95%UI)	1990 ASR per 100000 No.(95%UI)	2021ASR per 100000 No.(95%UI)	Percentage change (100%)	1990–2021 EAPC (95% UI)
China	3,869,629.14 (2,623,653.20, 5,322,757.05)	5,196,463.72 (3,572,714.95, 7,053,904.82)	330.26 (227.84, 451.47)	287.48 (200.08, 394.39)	-12.95 (-18.37, -6.85)	-0.65 (-0.83, -0.48)
India	4,186,259.36 (2,857,372.85, 5,744,847.61)	9,076,408.73 (6,164,809.04, 12,375,754.23)	601.67 (409.30, 818.53)	631.82 (429.57, 857.26)	5.01 (0.09, 10.56)	-0.86 (-1.20, -0.52)
United States of America	1,390,704.35 (965,054.21, 1,907,296.79)	3,011,988.34 (2,109,168.20, 4,042,178.69)	516.08 (358.97, 710.81)	899.32 (628.39, 1222.59)	74.26 (65.66, 83.39)	0.87 (0.52, 1.23)

ASR, Age-Standardized Rate; EAPC, Estimated Annual Percentage Change; UI, Uncertainty Intervals. The values represent the number of cases of Major Depressive Disorder (MDD) in 1990 and 2021, along with the corresponding ASR per 100,000 and the percentage change from 1990 to 2021, including the EAPC with 95% uncertainty intervals.

### Time trends

**Figure 1:**
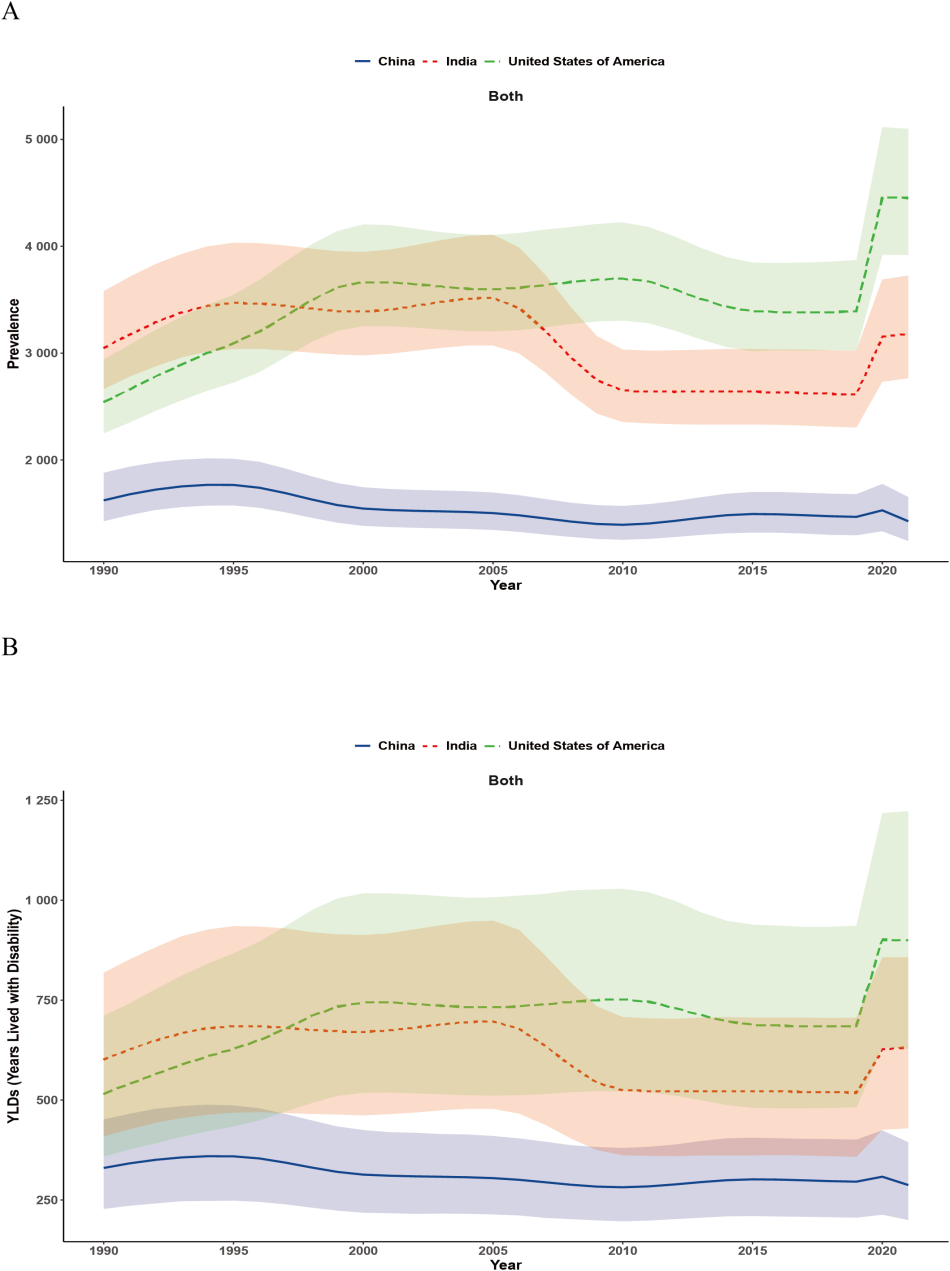
Trends in age-standardized prevalence rates (ASPR) and age-standardized years lived with disability rates (ASYR) of major depressive disorder (MDD) from 1990 to 2021 in China, India, and the United States of America. **(A)** ASPR; **(B)** ASYR. MDD, major depressive disorder; ASPR, age-standardized prevalence rate; ASYR, age-standardized years lived with disability.

From 1990 to 2021, MDD trends showed notable cross-country differences. In China, ASPR initially increased but then declined, while in India, the ASPR rose until the mid-2000s, then declined and stabilized. In contrast, the United States of America showed a steady rise until 2010, followed by a slight decrease. All three countries exhibited an abnormal peak in 2020 (**Figure 1A**), with the United States of America and India consistently higher than China. The ASYR followed similar patterns, with the United States of America maintaining the highest burden, China showing a decline followed by stabilization, and India fluctuating but remaining higher than China (**Figure 1B**).

### Age and gender differences

**Figure 2:**
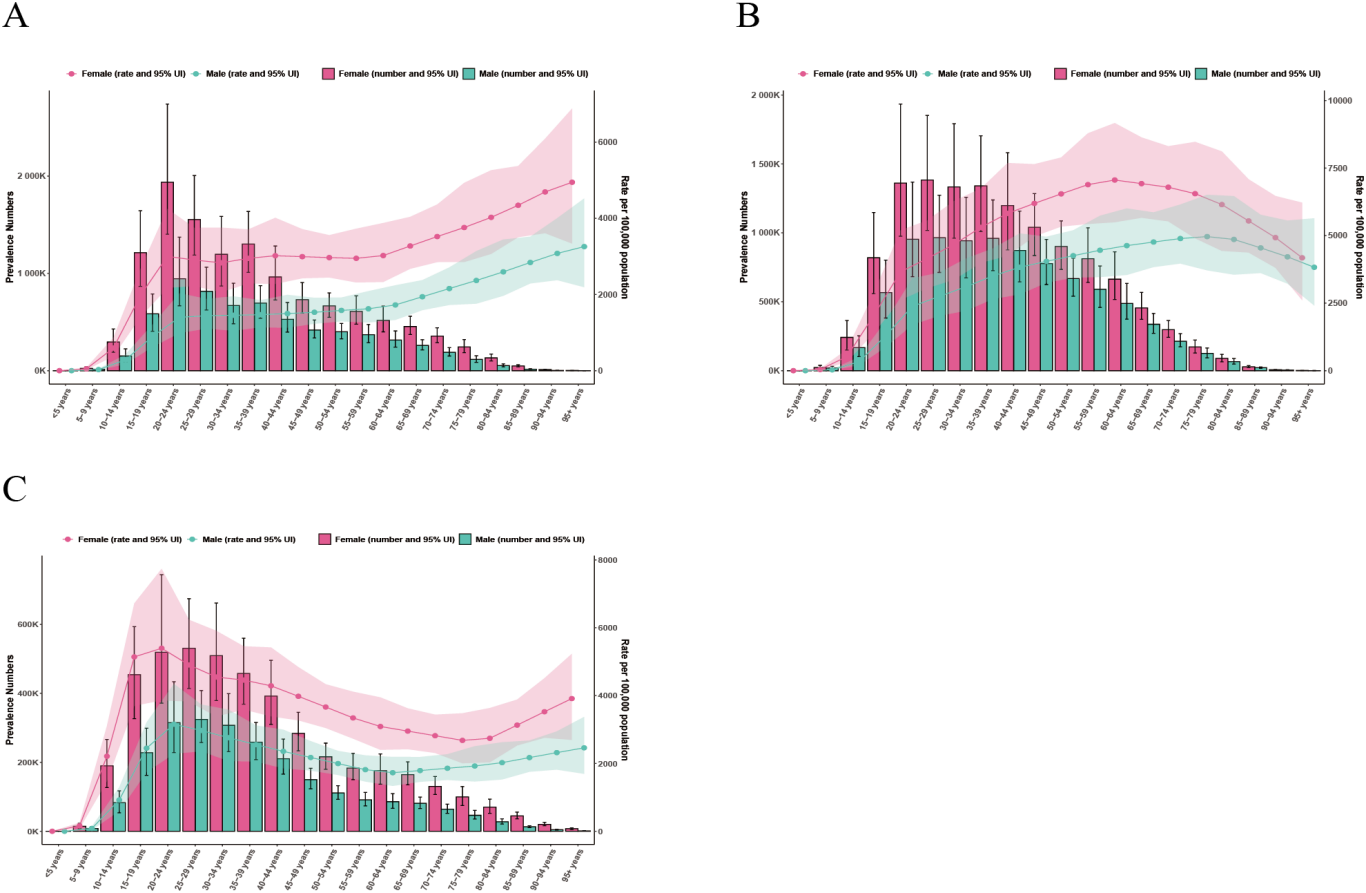
Age- and gender-specific prevalence rates of major depressive disorder (MDD) in China, India, and the United States of America in 1990. **(A)** China; **(B)** India; **(C)** The United States.

**Figure 3:**
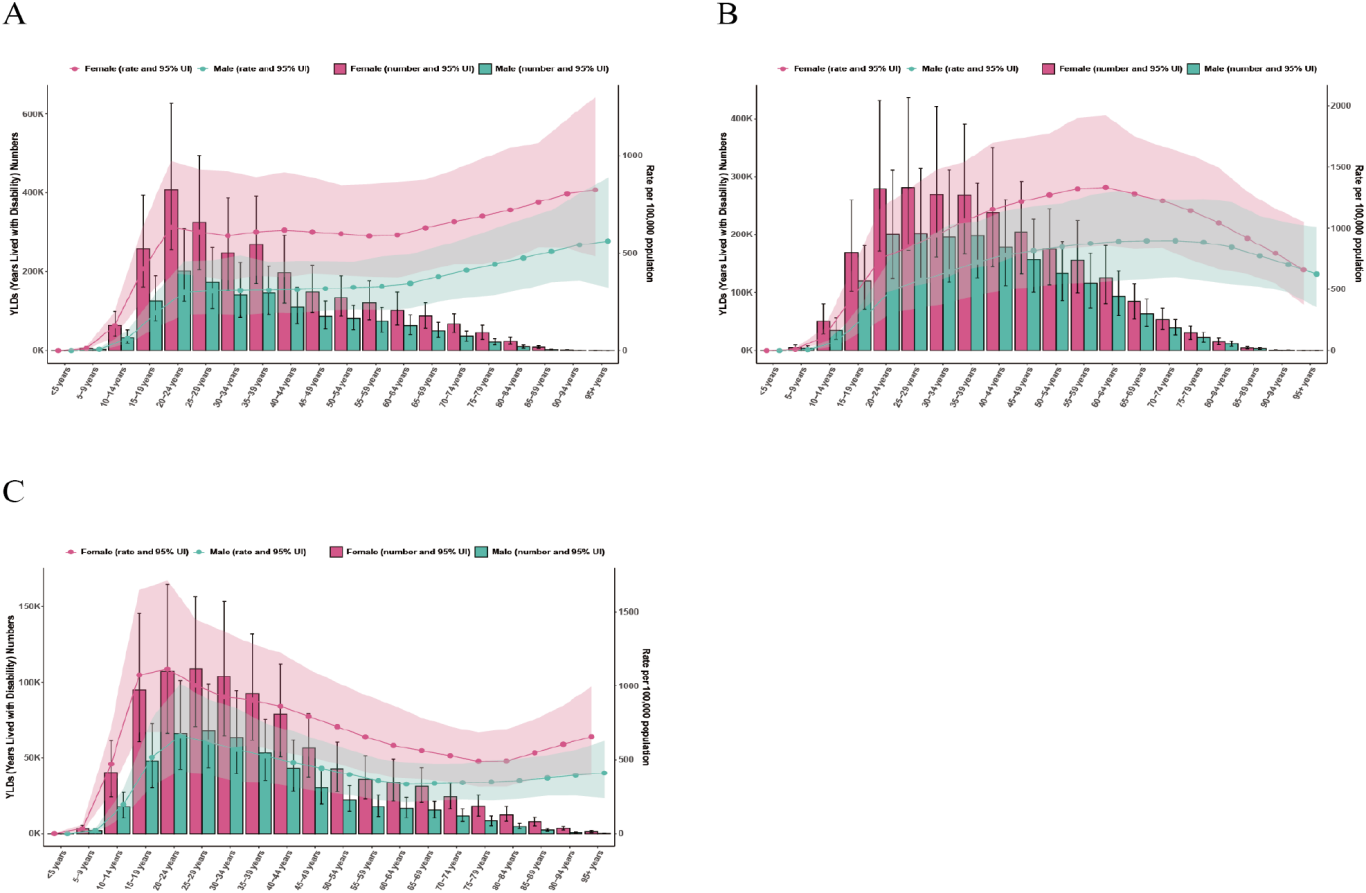
Age- and gender-specific years lived with disability (YLDs) of major depressive disorder (MDD) in China, India, and the United States of America in 1990. **(A)** China; **(B)** India; **(C)** The United States.

**Figure 4:**
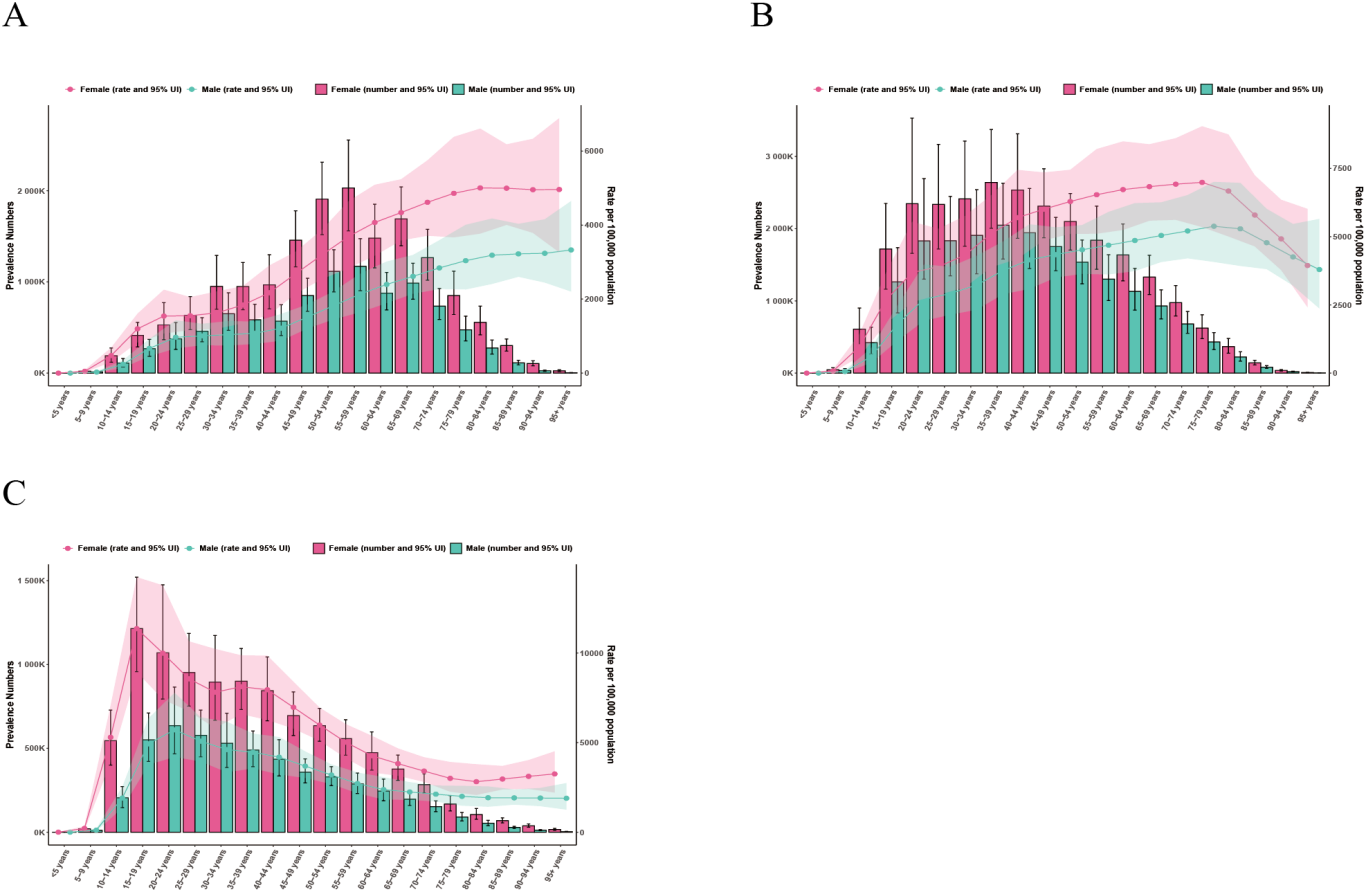
Age- and gender-specific prevalence rates of major depressive disorder (MDD) in China, India, and the United States of America in 2021. **(A)** China; **(B)** India; **(C)** The United States.

**Figure 5:**
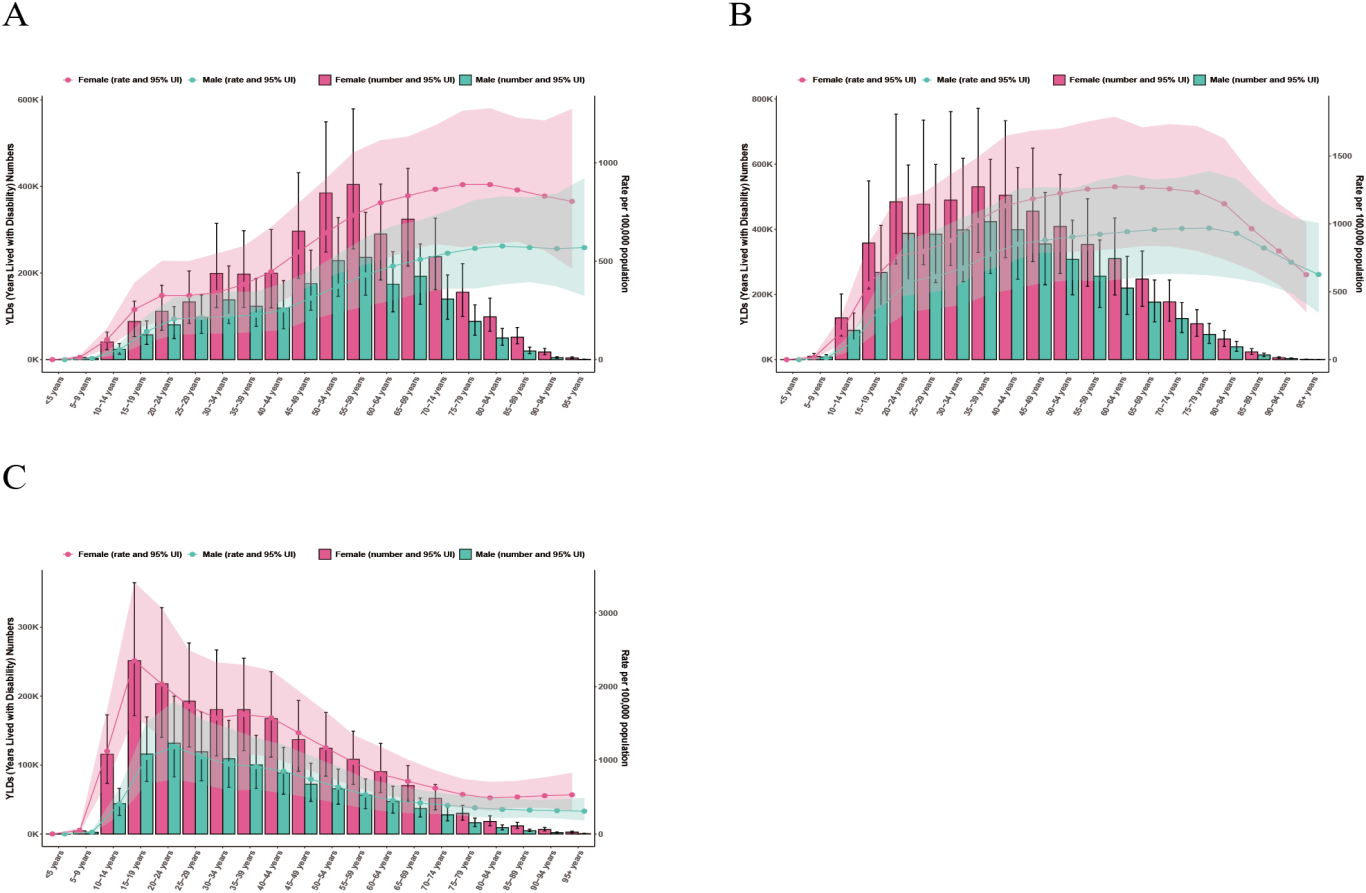
Age- and gender-specific years lived with disability (YLDs) of major depressive disorder (MDD) in China, India, and the United States of America in 2021. **(A)** China; **(B)** India; **(C)** The United States.

In both 1990 and 2021, females had higher prevalence and YLDs than males across all age groups in China, India, and the United States of America (**Figures 2**–**5**). In 1990, the highest prevalence and YLDs in China were seen in those aged 20–24 (**Figure 2A**), while in India and the United States, they peaked in the 25–29 age group (**Figures 2B**, **3C**). By 2021, the highest prevalence and YLDs shifted to older age groups in China and India, with China showing the highest rates in those aged 55–59 and India in those aged 35–39. In the United States of America, the highest prevalence and YLDs were observed in the 15–19 age group.

### Joinpoint regression results

**Figure 6:**
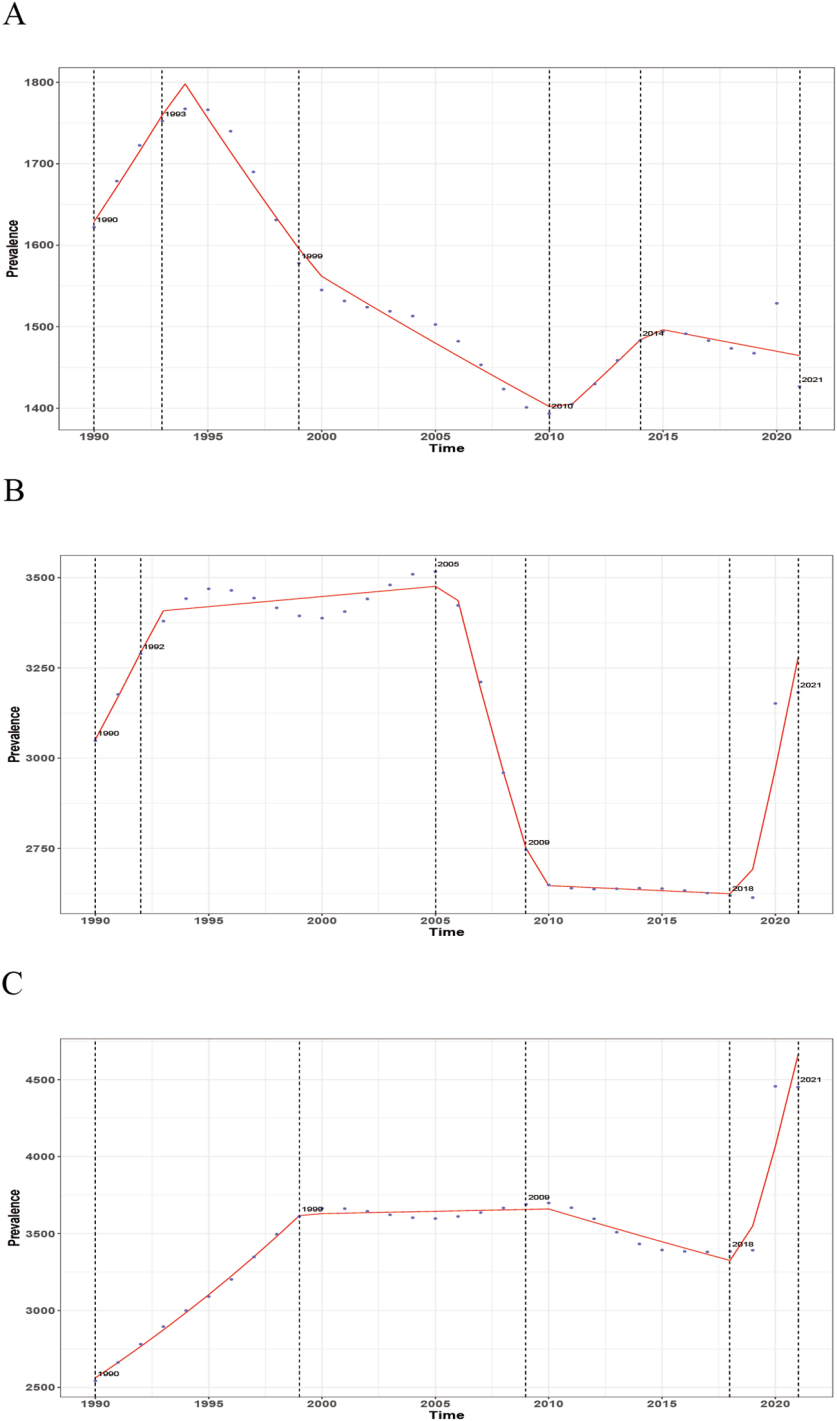
Joinpoint regression analysis of age-standardized prevalence rates (ASPR) of major depressive disorder (MDD) in China, India, and the United States of America from 1990 to 2021. **(A)** China; **(B)** India; **(C)** The United States.

From 1990 to 2021, the ASPR of MDD trends differed across China, India, and the United States of America. In China, the prevalence generally declined after an initial increase in the early 1990s, with a brief rise after 2010. In India, there were fluctuations in ASPR, with a decline from 2000 to 2010, followed by a rebound post-2015. The United States of America showed a consistent upward trend, with a sharp rise after 2020, despite a brief decline in the 2010s (**Figures 6**, **Supplementary Table S1**). Regarding MDD-related ASYR, China exhibited an overall decline, especially after the 1990s. In India, the trend was less consistent, with a decrease followed by an increase after 2015. The United States of America showed a steady increase, with a significant rise after 2019 (**Supplementary Figure S1**, **Supplementary Table S2**).

### Age−period−cohort analysis results

**Figure 7:**
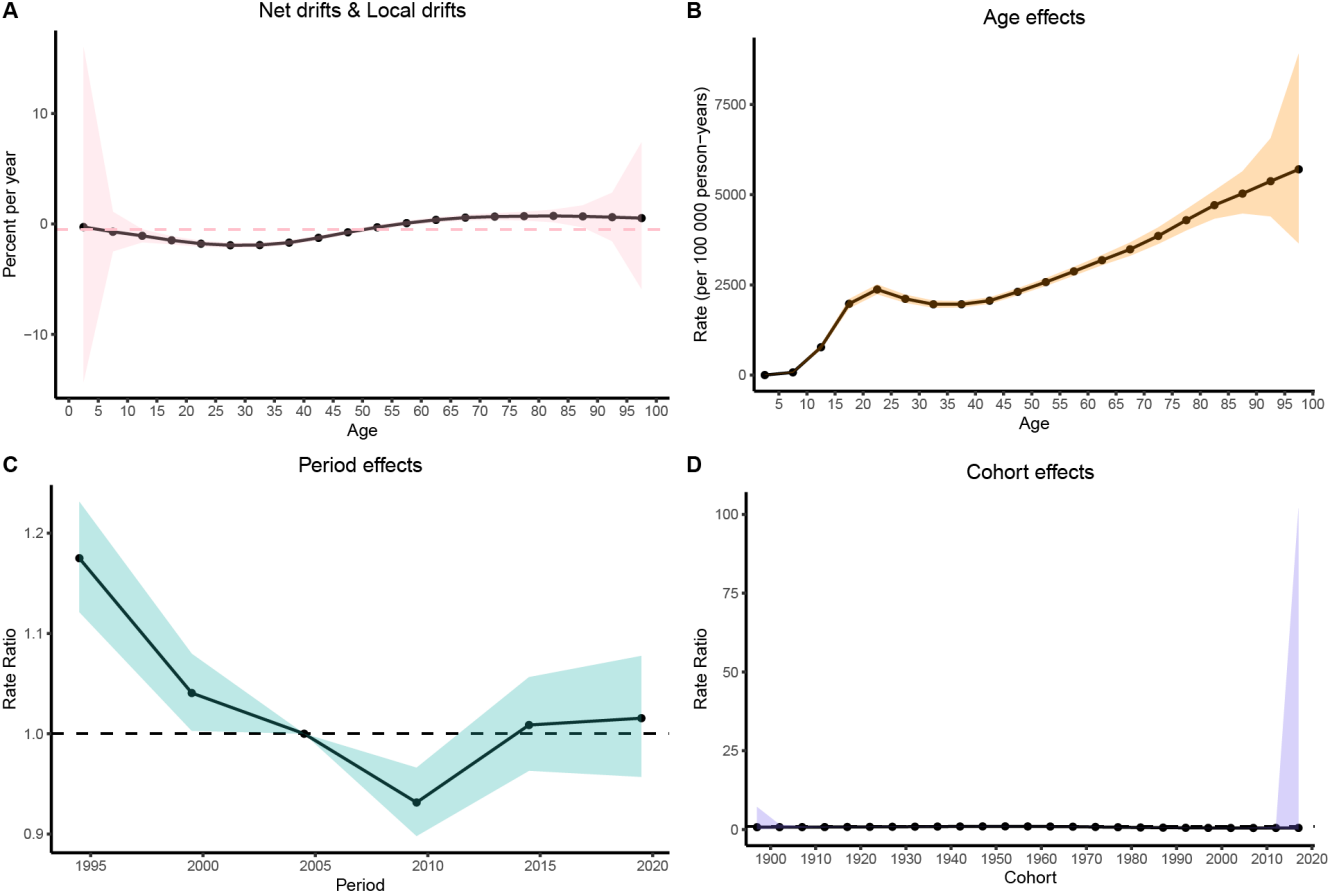
Age-period-cohort (APC) analysis of age-standardized prevalence rates (ASPR) of major depressive disorder (MDD) in China from 1990 to 2021. **(A)** Net drifts & Local drifts; **(B)** Age effects; **(C)** Period effects; **(D)** Cohort effects.

**Figure 8:**
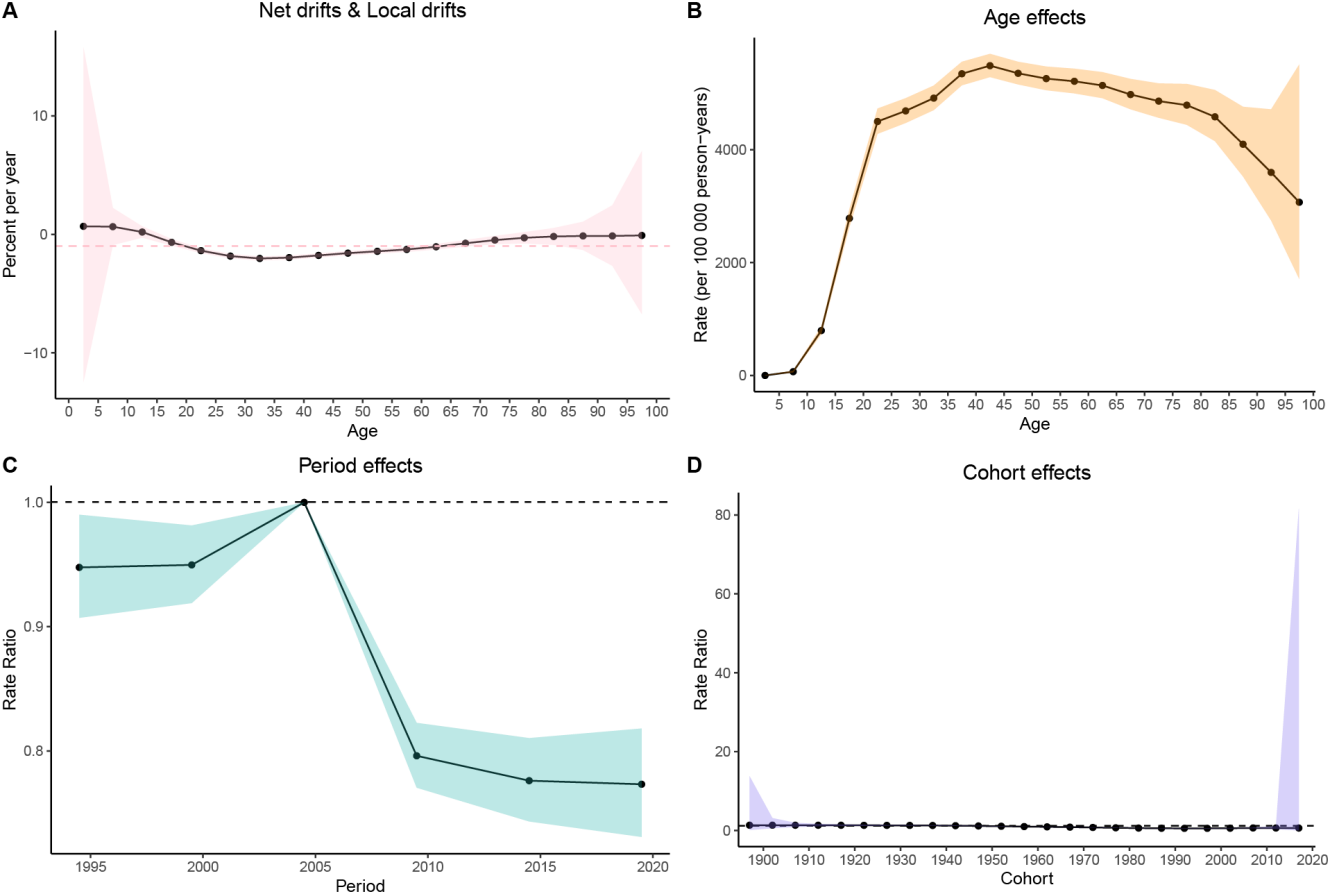
Age-period-cohort (APC) analysis of age-standardized prevalence rates (ASPR) of major depressive disorder (MDD) in India from 1990 to 2021. **(A)** Net drifts & Local drifts; **(B)** Age effects; **(C)** Period effects; **(D)** Cohort effects.

**Figure 9:**
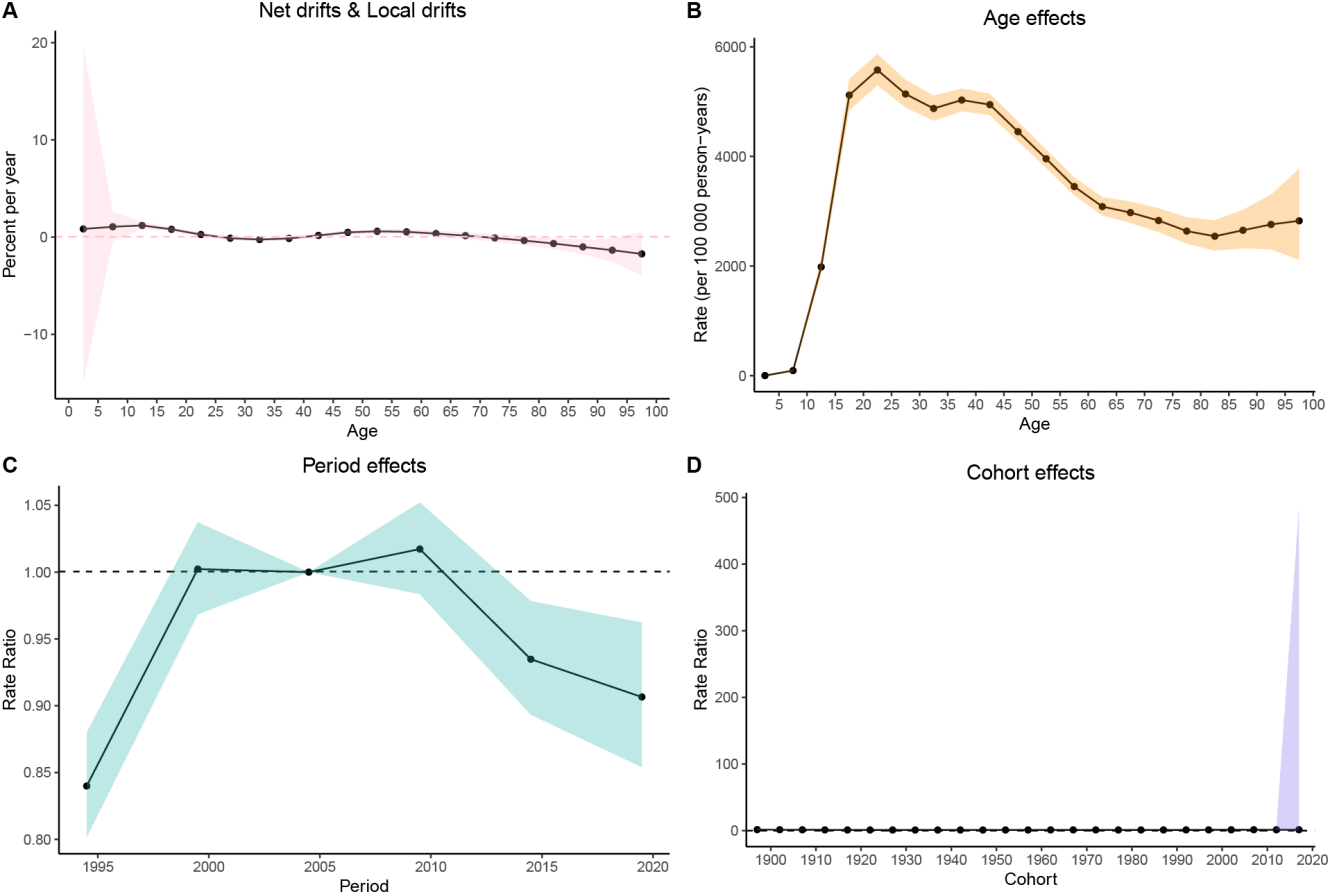
Age-period-cohort (APC) analysis of age-standardized prevalence rates (ASPR) of major depressive disorder (MDD) in the United States of America from 1990 to 2021. **(A)** Net drifts & Local drifts; **(B)** Age effects; **(C)** Period effects; **(D)** Cohort effects.

The age-period-cohort (APC) model was used to assess the independent effects of age, period, and cohort on the MDD prevalence in China, India, and the United States of America. In China, MDD prevalence increased during adolescence, peaked in young adulthood, and then rose again in older age groups (**Figure 7**). In India, the prevalence followed an inverted U-shaped curve, peaking in middle age before declining in older age groups (**Figure 8**). The United States of America showed a bimodal distribution, with peaks in early adulthood and older age groups (**Figure 9**).

Regarding the period effect, prevalence in China stabilized after 2014 (**Figure 7**). India experienced a decline after 2009 (**Figure 8**), while in the United States of America, prevalence fluctuated slightly before declining after 2010 (**Figure 9**). The cohort effect revealed divergent generational trends: In China, younger cohorts showed decreasing risks (**Figure 7**), whereas in India, risks decreased until the 1970s cohorts before rebounding in more recent generations (**Figure 8**). In contrast, the United States of America exhibited a U-shaped pattern, with risks declining before 1957 and increasing steadily thereafter (**Figure 9**).

The net drift analysis indicated a decline in both China (–0.51%/year, 95% CI: –0.86% to –0.16%) and India (–1.05%/year, 95% CI: –1.39% to –0.71%), while the United States of America remained largely stable (+0.11%/year, 95% CI: –0.25% to 0.47%). Local drifts analysis further suggested decreasing trends in younger and middle-aged groups in China and India, while in the United States of America, adolescents showed an increasing trend (**Figures 7**, **9**).

### Risk factors

**Figure 10:**
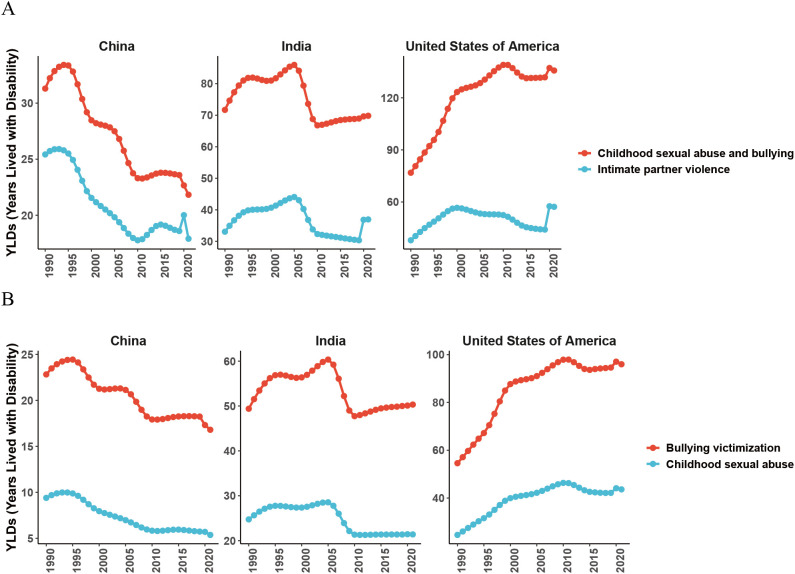
Risk factors contributing to major depressive disorder (MDD) in China, India, and the United States of America from 1990 to 2021. **(A)** Level 2 risk factors, including childhood sexual abuse and bullying, and intimate partner violence, and their relative contributions to MDD in China, India, and the United States of America. **(B)** Level 3 risk factors, specifically bullying victimization and childhood sexual abuse, and their relative contributions to MDD in China, India, and the United States of America.

The GBD 2021 database comprehensively evaluated the impact of 88 risk factors on global health outcomes, categorized these factors into four levels. The first level includes three broad categories: environmental/occupational risks, behavioral risks, and metabolic risks. The subsequent risk factors were further subdivided based on first-level risk factors. In this study, the identified MDD risk factors were classified as follows: level 1 risk factors included behavioral risks; level 2 encompassed childhood sexual abuse and bullying, intimate partner violence; and level 3 specifically highlighted bullying victimization and childhood sexual abuse. From 1990 to 2021, the top risk factors among the level 2 risk factors in China, India and the United States of America were child sexual abuse and bullying, followed by intimate partner violence, as shown in **Figure 10A**. For level 3, the primary risk factor was bullying victimization, followed by child sexual abuse, as shown in **Figure 10B**.

### Future forecasts of the ASPR of MDD

**Figure 11:**
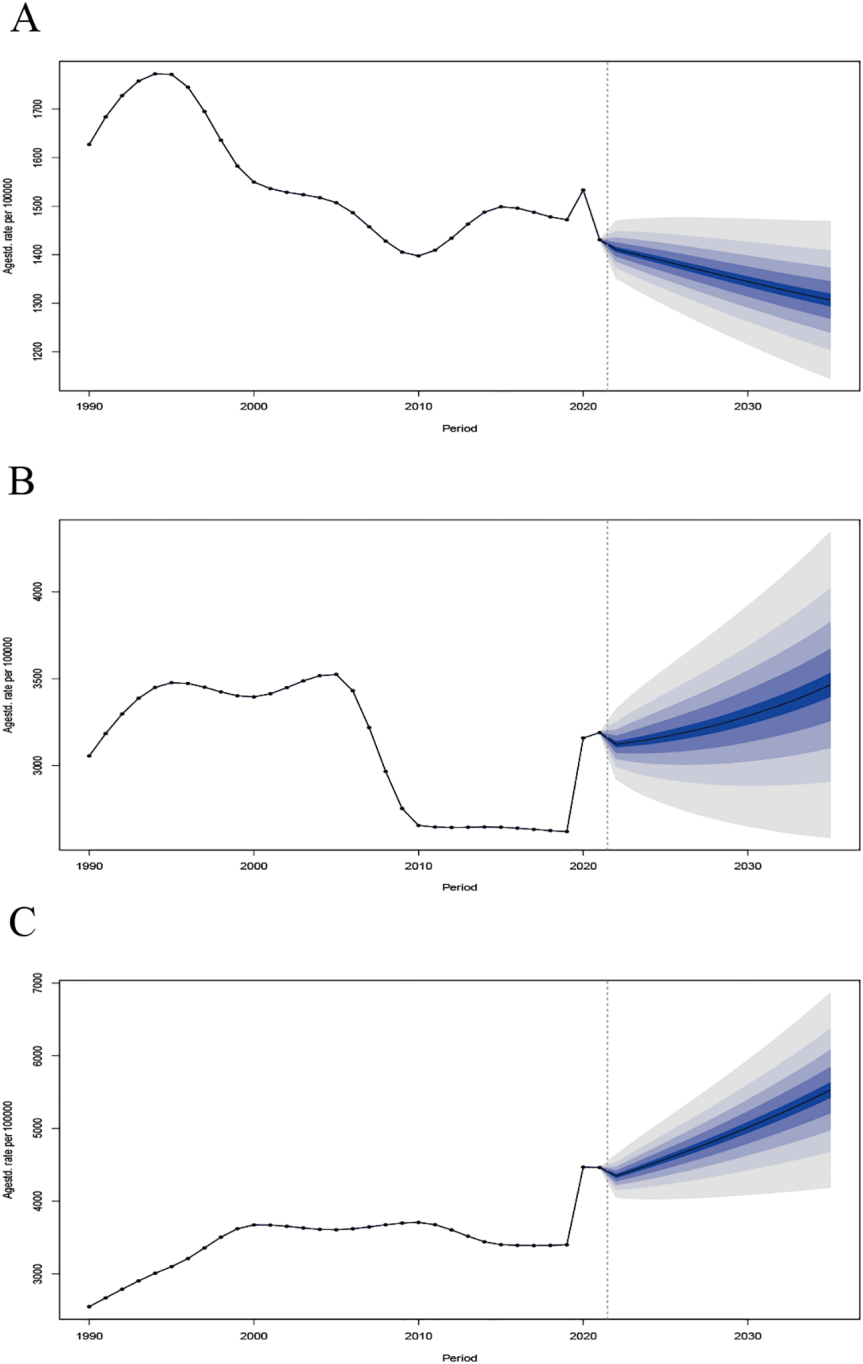
Forecast of age-standardized prevalence rates (ASPR) of major depressive disorder (MDD) in China, India, and the United States of America from 2022 to 2035. **(A)** China; **(B)** India; **(C)** The United States of America.

Over the next decade (2022–2035), the ASPR of MDD is projected to show a slight decrease in China, remain relatively stable in India, and increase moderately in the United States of America. In China, the ASPR is estimated to be 1,410.72 per 100,000 people in 2022 (95% CI: 1,365.78–1,489.90), and it is projected to slightly decrease to 1,306.74 per 100,000 people in 2035 (95% CI; 1,113.80–1,499.67). In India, the ASPR is expected to increase slightly, from 3,122.49 per 100,000 in 2022 (95% CI: 2,881.27–3,363.70) to 3,463.87, per 100,000 by 2035 (95% CI: 2,415.29–4512.45). In contrast, the United States of America is projected to experience a moderate increase, from 4,346.06 per 100,000 in 2022 (95% CI: 3,995.73–4,696.39) to 5,526.14, per 100,000 in 2035 (95% CI: 3,933.81–7,118.48) (**Figure 11**).

## Discussion

The study provides the first systematic, long-term comparison of the burden of MDD in China, India and the United States of America using GBD 2021 data. Over the past three decades, the absolute number of MDD cases and YLDs increased in all three countries. However, age-standardized trends diverged: In China, both ASPR and ASYR modestly declined; While in India, they remained stable; and in the United States of America, both indicators rose significantly. Projections to 2035 suggest that ASPR will remain stable in China and India, while it is projected to continue rising in the United States, indicating a persistent and increasing MDD burden in this high-income country despite improvements in mental health care and awareness.

The burden of MDD showed distinct patterns across China, India, and the United States of America. In China, MDD cases increased mainly due to demographic changes, such as population growth and aging, with ASRs declining modestly, suggesting that changes in age-specific risk were less significant. In India, MDD cases rose but ASRs remained stable, reflecting the combined effects of rapid population growth and limited mental health resources, particularly in rural areas (16). In the United States, both case numbers and ASRs increased sharply, reflecting a genuine rise in age-specific risk, probably worsened by socioeconomic inequality and greater recognition of mental health issues (17). These findings highlight the need for targeted mental health interventions that address both demographic changes and evolving social determinants across countries, similar to the challenges observed in disorders such as obsessive-compulsive disorder (18).

Joinpoint regression revealed divergent temporal patterns in MDD burden across the three countries. In China, a brief rise in MDD cases in the early 1990s was followed by a decline and stabilization, likely reflecting improvements in healthcare and mental health policy (19). In India, a decrease in ASRs in the 2000s was reversed in recent years, indicating that early gains in mental health services struggled to keep pace with rapid urbanization and rising societal pressures (16). In the United States of America, MDD rates consistently increased, with a sharp surge after 2019–2020, coinciding with the COVID-19 pandemic. Although causality cannot be determined, the timing of this increase aligns with global evidence of pandemic-related mental health deterioration, particularly in the United States of America, where higher baseline prevalence and socioeconomic disruptions likely amplified the impact (20).

Our findings confirm persistent sex differences in MDD burden, as women consistently experiencing higher prevalence and YLDs than men in China, India, and the United States, reflecting a complex mix of biological, psychological, and socio-cultural factors, such as gender-based violence and social role strain (21). Cultural stigma and gender norms further influence help-seeking behaviors, with women more likely to seek mental health support (22). Over time, the age distribution of MDD burden shifted, with China seeing higher prevalence among older adults, India in midlife, and the United States in younger age groups. These shifts suggest that cohort-specific exposures and life-course transitions are altering age-specific risks (23, 24). In high-income settings, younger cohorts are affected by pressures related to education, employment, and digital media, while in middle-income settings, chronic diseases and financial strain predominantly affect older and middle-aged adults (25–27).

The APC analysis shows that MDD risk is increasingly shaped by birth cohort and social context rather than age alone. In China, declining risks in recent cohorts align with improvements in early-life conditions, education, and social protection. In India, rising risks in younger cohorts reflect psychosocial stress amid rapid societal change (28). Especially among adolescents, increased risks in recent cohorts signal growing vulnerability in the United States of America, consistent with rising depressive symptoms and suicide risk in youth (29). Period effects were modest, with improvements in China’s mental health system after 2014, but short-term declines in India and the United States of America were insufficient to counteract negative cohort trends, especially during the COVID-19 pandemic. These findings suggest that cohort-targeted, youth-focused strategies are crucial, as broader social factors often limit the impact of policy alone.

Given these trends, targeted interventions are critical. **Table 3** summarizes the policy priorities across China, India, and the United States, which offer tailored intervention strategies to address the unique mental health challenges in each country. In China, the increasing mental health burden among older adults highlights the need for improved services, including early detection and community-based care (30). In India, youth-focused interventions should focus on improving mental health literacy, addressing psychosocial pressures, and expanding community resources. Strengthening social support networks and providing career guidance can help mitigate the psychological stress associated with rapid societal change, particularly in rural areas where service gaps are more pronounced (31). In the United States of America, youth-focused strategies should emphasize mental health education, early screening, and enhanced social support to address the rising burden among adolescents and young adults (32).

**Table 3:** Policy priorities for reducing major depressive disorder (MDD) burden in China, India, and the United States of America.

Location	Disease burden trends	Priority population	Key public health interventions
China	Aging-driven burden with declining age-standardized rates	Older adults	• Expand community-based mental health services • Strengthen early detection of late-life depression • Integrate mental health into primary and geriatric care
India	Stable age-standardized rates with rising absolute burden	Adolescents & young adults	• Improve mental health literacy •Address psychosocial stressors linked to rapid social change •Expand community and school-based services, especially in rural areas
United States of America	Increasing age-specific risk, especially post-2019	Adolescents & young adults	• Implement school-based mental health education • Strengthen early screening and referral systems • Enhance social support and access to care

Our risk-factor analysis highlights the importance of early-life and interpersonal adversities in shaping the MDD burden. Across China, India, and the United States, childhood sexual abuse, bullying, and intimate partner violence emerged as major contributors, consistent with evidence linking these exposures to increased risk of depression across the life course (33, 34). Such adversities may also impair metacognitive functioning, including difficulties in regulating cognitive and emotional processes, which reduces adaptive coping and amplifying the overall burden of MDD (35). Broader societal stressors, including urbanization, labor migration, and economic insecurity, are likely to further exacerbate MDD burden by increasing psychosocial stress and limiting access to care (36–38). However, while the the GBD framework is model-based and cannot establish causality, the consistency between our findings and prior research suggests that reducing violence and victimization and strengthening support for affected individuals could help mitigate the long-term burden of MDD in all three countries (39).

BAPC projections suggest that ASPR of MDD will slightly decrease in China, remain relatively stable in India, and rise in the United States of America. In both China and India, this stability reflects the impact of population aging. This will likely increase the number of people living with MDD, underscoring the need to address treatment gaps, enhance community-based services, and integrate mental health interventions into primary care (40). Although ASRs have modestly declined in China, the growing burden in both countries highlights disparities between the growing population and limited healthcare resources, especially in rural and underserved areas (41, 42). In the United States of America, the increasing trend in MDD suggests a deepening mental health crisis that requires not only targeted interventions but also efforts to address socioeconomic disparities and access to care (43). Additionally, the long-term psychological impact of the COVID-19 pandemic, including grief and trauma, remains a critical consideration for future mental health planning (44).

This study has several limitations. First, the GBD estimates for MDD are derived from epidemiological surveys, clinical records, and statistical modelling. In many low- and middle-income settings, mental health surveillance remains incomplete, and underdiagnosis, particularly of mild, subthreshold and untreated cases, is likely. This may lead to an underestimation of the true burden. Second, cross-country and temporal differences in diagnostic criteria, screening tools and cultural expressions of psychological distress may affect comparability between China, India and the United States of America, even after standardization. Additionally, the ecological nature of our country-level comparisons limits the ability to draw individual-level conclusions, as differences in healthcare systems, policies, and social contexts may confound these findings. Third, the model-based risk-attribution framework simplifies the complex interplay of determinants such as genetic vulnerability, early-life adversity, socioeconomic disadvantage and physical comorbidity. The framework primarily captures direct burden (e.g., prevalence and YLDs) while underrepresenting indirect consequences, including caregiver strain and educational or occupational losses. Finally, projections are extrapolated from past trends and existing data, which may not fully account for rapid changes in policy, service delivery, social conditions or emerging interventions. As a result, forecasts of future MDD trajectories should be interpreted with caution.

## Conclusion

The burden of MDD varies significantly across China, India, and the United States of America, reflecting the diverse socio-economic, cultural, and healthcare contexts in each country. While China and India have experienced more stable trends in MDD prevalence and YLDs, the United States of America has seen a pronounced increase in both, highlighting the growing public health challenge in high-income countries. Gender disparities were evident in all three countries, with women consistently bearing a higher burden than men. Despite these differences, all three countries face common challenges in addressing MDD, particularly in the context of aging populations, socio-economic stressors, and healthcare access. Projections suggest that while the MDD burden in China and India may stabilize, the United States of America is likely to experience a continued rise in MDD prevalence. In response, targeted mental health strategies, including improving access to care, addressing socio-economic inequalities, and strengthening prevention and intervention efforts, are essential to mitigate the future impact of MDD in these countries.

## Data Availability

Publicly available datasets were analyzed in this study. This data can be found here: https://ghdx.healthdata.org/gbd-2021.

## Supplementary material

Supplementary Figure 1 Joinpoint Regression Analysis of Age-Standardized Years Lived with Disability Rates (ASYR) of Major Depressive Disorder (MDD) in China, India, and the United States of America from 1990 to 2021. **(A)** China; **(B)** India; **(C)** The United States of America.
