# Supplementary figures and images for: Cross−national comparison of major depressive disorder burden in China, India, and the United States of America: an age−period−cohort analysis of GBD 2021
Source: Front Psychiatry. 2026 Jan 20;16:1686919. doi: 10.3389/fpsyt.2025.1686919 (PMC12864386; doi:10.3389/fpsyt.2025.1686919)

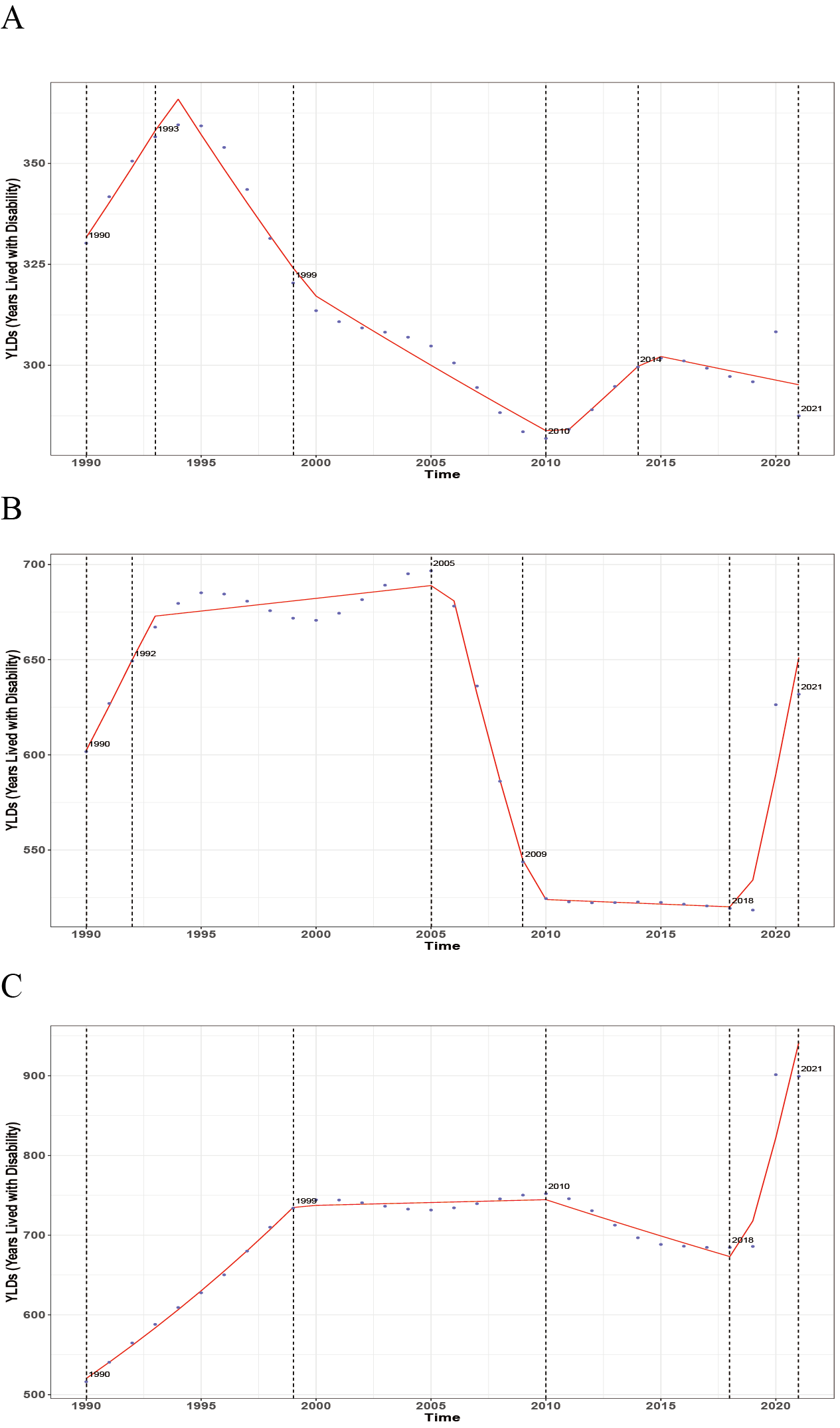

Supplement: Supplementary Figure 1 — Joinpoint Regression Analysis of Age-Standardized Years Lived with Disability Rates (ASYR) of Major Depressive Disorder (MDD) in China, India, and the United States of America from 1990 to 2021. (A) China; (B) India; (C) The United States of America. [file Image1.tif]
