# Supplementary material for: Cross−national comparison of major depressive disorder burden in China, India, and the United States of America: an age−period−cohort analysis of GBD 2021
Source: Front Psychiatry. 2026 Jan 20;16:1686919. doi: 10.3389/fpsyt.2025.1686919 (PMC12864386; doi:10.3389/fpsyt.2025.1686919)
Supplement: Supplementary file 2 [file Table1.docx]

**Supplementary Materials**

**Supplementary Table S1**

Joinpoint regression analysis of prevalence rates (per 100,000 population) for major depressive disorder in China, India, and the United States of America, 1990–2021.

| **Location** | **Period** | **APC (95% CI)** | **p-value** |
| --- | --- | --- | --- |
| China | 1990-1993 | 2.61 (1.39 - 3.85) | ＜0.01 |
|  | 1993-1999 | -2.36 (-2.98 - -1.73) | 2.86 |
|  | 1999-2010 | -1.07 (-1.33 - -0.82) | 2.22 |
|  | 2010-2014 | 1.84 (0.63 - 3.07) | ＜0.01 |
|  | 2014-2021 | -0.36 (-0.86 - 0.15) | 0.16 |
| AAPC | 1990-2021 | -0.43 (-0.70 - -0.15) | ＜0.01 |
| India | 1990-1992 | 3.88 (1.37 - 6.45) | ＜0.01 |
|  | 1992-2005 | 0.16 (-0.09 - 0.42) | 0.21 |
|  | 2005-2009 | -7.16 (-8.58 - -5.71) | ＜0.01 |
|  | 2009-2018 | -0.11 (-0.55 - 0.34) | 0.64 |
|  | 2018-2021 | 10.34 (7.67 - 13.07) | 3.11 |
| AAPC | 1990-2021 | 0.36 (-0.04 - 0.77) | 0.07 |
| United States of America | 1990-1999 | 3.90 (3.35 - 4.46) | ＜0.01 |
|  | 1999-2009 | 0.08 (-0.45 - 0.62) | 0.76 |
|  | 2009-2018 | -1.19 (-1.81 - -0.57) | ＜0.01 |
|  | 2018-2021 | 14.59 (10.73 - 18.59) | 7.33 |
| AAPC | 1990-2021 | 2.23 (1.74 - 2.72) | ＜0.01 |

Note: APC: annual percent change; CI: confidence interval

**Supplementary Table S2**

Joinpoint regression analysis of YLD rates (per 100,000 population) for major depressive disorder in China, India, and the United States of America, 1990–2021.

| **Segments** | **Period** | **APC (95% CI)** | **p-value** |
| --- | --- | --- | --- |
| China | 1990-1993 | 2.59 (1.35 - 3.85) | ＜0.01 |
|  | 1993-1999 | -2.40 (-3.03 - -1.76) | 3.00 |
|  | 1999-2010 | -1.10 (-1.36 - -0.85) | ＜0.01 |
|  | 2010-2014 | 1.80 (0.57 - 3.05) | ＜0.01 |
|  | 2014-2021 | -0.39 (-0.90 - 0.13) | 0.14 |
| AAPC | 1990-2021 | -0.46 (-0.74 - -0.18) | ＜0.01 |
| India | 1990-1992 | 3.88 (1.35 - 6.47) | ＜0.01 |
|  | 1992-2005 | 0.20 (-0.06 - 0.46) | 0.14 |
|  | 2005-2009 | -7.18 (-8.61 - -5.72) | ＜0.01 |
|  | 2009-2018 | -0.09 (-0.54 - 0.36) | 0.69 |
|  | 2018-2021 | 10.40 (7.71 - 13.15) | 3.55 |
| AAPC | 1990-2021 | 0.39 ( -0.02 - 0.79) | 0.06 |
| United States of America | 1990-1999 | 3.91 (3.36 - 4.47) | ＜0.01 |
|  | 1999-2009 | 0.10 (-0.37 - 0.56) | 0.68 |
|  | 2009-2018 | -1.25 (-1.99 - -0.51) | ＜0.01 |
|  | 2018-2021 | 14.53 (10.66 - 18.54) | 9.99 |
| AAPC | 1990-2021 | 2.25 (1.76 - 2.74) | ＜0.01 |

Note: YLD, years lived with disability；APC: annual percent change; CI: confidence interval
